# Secreted protein acidic and rich in cysteine (SPARC) induces cell migration and epithelial mesenchymal transition through WNK1/snail in non-small cell lung cancer

**DOI:** 10.18632/oncotarget.19475

**Published:** 2017-07-22

**Authors:** Jen-Yu Hung, Meng-Chi Yen, Shu-Fang Jian, Cheng-Ying Wu, Wei-An Chang, Kuan-Ting Liu, Ya-Ling Hsu, Inn-Wen Chong, Po-Lin Kuo

**Affiliations:** ^1^ School of Medicine, College of Medicine, Kaohsiung Medical University, Kaohsiung, Taiwan; ^2^ Division of Pulmonary and Critical Care Medicine, Department of Internal Medicine, Kaohsiung Medical University Hospital, Kaohsiung, Taiwan; ^3^ Department of Emergency Medicine, Kaohsiung Medical University Hospital, Kaohsiung Medical University, Kaohsiung, Taiwan; ^4^ Graduate Institute of Clinical Medicine, College of Medicine, Kaohsiung Medical University, Kaohsiung, Taiwan; ^5^ Graduate Institute of Medicine, College of Medicine, Kaohsiung Medical University, Kaohsiung, Taiwan; ^6^ Department of Respiratory Therapy, College of Medicine, Kaohsiung Medical University, Kaohsiung, Taiwan; ^7^ Institute of Medical Science and Technology, National Sun Yat-Sen University, Kaohsiung, Taiwan

**Keywords:** SPARC, WNK1, lung cancer, EMT, migration

## Abstract

The extracellular matrix is a component of physiological microenvironment and a regulator of cellular processes such as migration and proliferation. Secreted Protein Acidic and Rich in Cysteine (SPARC/osteonectin) is an extracellular matrix-associated glycoprotein involved in the regulation of cell proliferation and cell migration in several types of cancers. However, the role of SPARC in lung cancer is paradoxical and details of the regulatory mechanism are not well-known. In this study, we investigated novel SPARC-mediated signaling pathways. Treatment of SPARC increased cell proliferation, migration, and mesenchymal phenotype in two non-small cell lung cancer cell lines, CL1-5 and H1299. We found that these phenotypes were not regulated by focal adhesion kinase and Src kinase, but were mediated by with no lysine (K) kinase 1 (WNK1). Suppression of WNK1 expression decreased the expression of SPARC-induced N-cadherin and smooth muscle actin. Moreover, Snail, an important transcription factor for regulating epithelial–mesenchymal transition, is also involved in SPARC/WNK1 pathway. In a murine tumor model, SPARC treatment significantly induced phosphorylation of Akt and WNK1 in lung tumor nodules when compared to control mice. In conclusion, these data suggest that WNK1 is a novel molecule in SPARC-mediated mesenchymal signaling pathway in non-small cell lung cancer.

## INTRODUCTION

Lung cancer is not only the most common cancer worldwide, but also the most common cause of cancer related deaths globally [1]. In the United States, lung cancer has been the leading cause of cancer related deaths for several decades, even though it is not the most common cancer [2]. Lung cancer is usually diagnosed at a late stage, and the overall survival rate for those patients of an advanced stage is poor, even with chemotherapy [3]. The development of targeting agents, such as epidermal growth factor receptor tyrosine kinase inhibitors (EGFR-TKIs) or EML4-ALK inhibitors, extends the survival of those patients with driver gene mutation [4]. Since 2015, three agents of immunotherapy, either PD-1 or PD-L1 antibodies, have been approved by the FDA to be used in the treatment of lung cancer patients, for they all showed some effect in prolonging the survival rates of lung cancer patients with or without driver gene mutation [5–9]. Unfortunately, not all lung cancer patients will show a response to target therapy or immunotherapy, and even if there is a response, the survival benefits are still limited. Therefore, identifying details of the tumorigenic signaling pathways and novel therapeutic targets is still an emerging issue.

SPARC (Secreted Protein, Acidic and Rich in Cysteine), also known as osteonectin or BM40, is a multifunctional glycoprotein which belongs to the matricellular protein family. Matricellular protein has been applied to a group of extracellular proteins with the function of modulating cell-matrix interactions and cell function [10]. SPARC itself is involved in the regulation of multiple biological processes, including cell proliferation and cell migration [11]. In different types of human cancers, SPARC plays either an oncogenic or tumor-suppressive role [12]. SPARC is secreted from cancer cells and neighboring stroma (tumor microenvironment) [13]. A high expression level of SPARC expression is associated with high metastatic potential of glioma and melanoma, and is furthermore associated with the poor prognosis of prostate cancer and breast cancers [14, 15]. However, SPARC is also reported to play a different role in breast and prostate cancers, with a tumor suppressive function [16, 17]. In lung cancer patients, high SPARC expression in lung stroma cells is associated with poor prognosis [18]. SPARC is essential for Snail-driven invasion through activation of mitogen-activated protein (MAP) kinase pathways in non-small cell lung cancer pathogenesis [19]. On the other hand, negative expression of SPARC in lung cancer cells indicates a poor prognosis for the overall survival of these patients [20]. It is paradoxical that lung cancer stroma cells expressing SPARC are associated with poor prognoses and lung cancer-expressing SPARC is associated with good prognoses. This evidence might imply that stroma cell-secreted SPARC plays a different role from SPARC in lung cancer. Further investigation of the downstream signaling pathways in lung cancer might clarify this puzzle.

Previous studies have indicated that SPARC promotes bone metastasis and epithelial-mesenchymal transition (EMT) in some types of highly metastatic cancers, including melanoma, prostate cancer, and breast cancer [21, 22]. Snail, an important transcription factor of EMT program, induces SPARC expression in non-small cell lung cancer [19], but the SPARC-mediated signaling pathways are not fully understood. This study revealed that SPARC activated with no lysine (K) kinase 1 (WNK1) and its signaling pathways in lung cancer cells, and in a murine tumor model.

## RESULTS

### SPARC treatment promotes migration and EMT phenotype in non-small cell lung cancer cells

**Figure 1:**
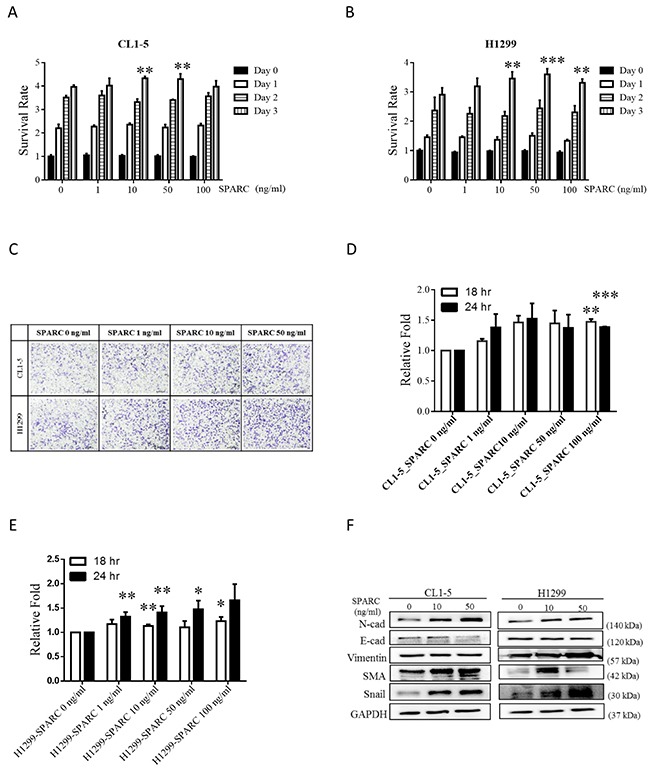
SPARC stimulation induces proliferation, migration and EMT signaling pathways in lung cancer cells CL1-5 or H1299 cells were seeded into 96 wells, and the proliferation rate was determined after SPARC stimulation at concentrations of 0, 10, 50, and 100 ng/mL. The proliferation rates of **(A)** CL1-5 and **(B)** H1299 cells are shown. **(C)** Cell migration was determined by transwell migration assay. The representative image is one of the images taken from the bottom surface. The number of cells in four random microscopic fields were counted. The number of **(D)** CL1-5 and **(E)** H1299 cells is shown. **(F)** Western blot assay showed protein expression levels of EMT signaling pathways. The error bars represent SD (t-test; * p < 0.05, ** p < 0.01, *** p < 0.001).

Recent studies suggest a relationship between SPARC and EMT program in highly metastatic cancers [21]. Therefore, two non-small cell lung cancer cell lines, CL1-5 and H1299, with highly metastatic activity were chosen. In Figure 1A and 1B, the proliferation rates of CL1-5 and H1299 were significantly enhanced after recombinant SPARC treatment at concentrations of 10 and 50 ng/mL. 100 ng/mL and 1-100 ng/mL SPARC treatment ehnahced migration rates of CL1-5 and H1299 cells (Figure 1C-1E). The mesenchymal phenotype-associated molecules, including N-cadherin, smooth muscle actin and Snail in CL1-5, and N-cadherin, Vimentin, and Snail in H1299 were induced by SPARC (Figure 1F). These results indicate that exogenous SPARC plays an oncogenic role in highly metastatic lung cancer cells.

### FAK/Src pathway might not be the critical regulator in SPARC-induced EMT

**Figure 2:**
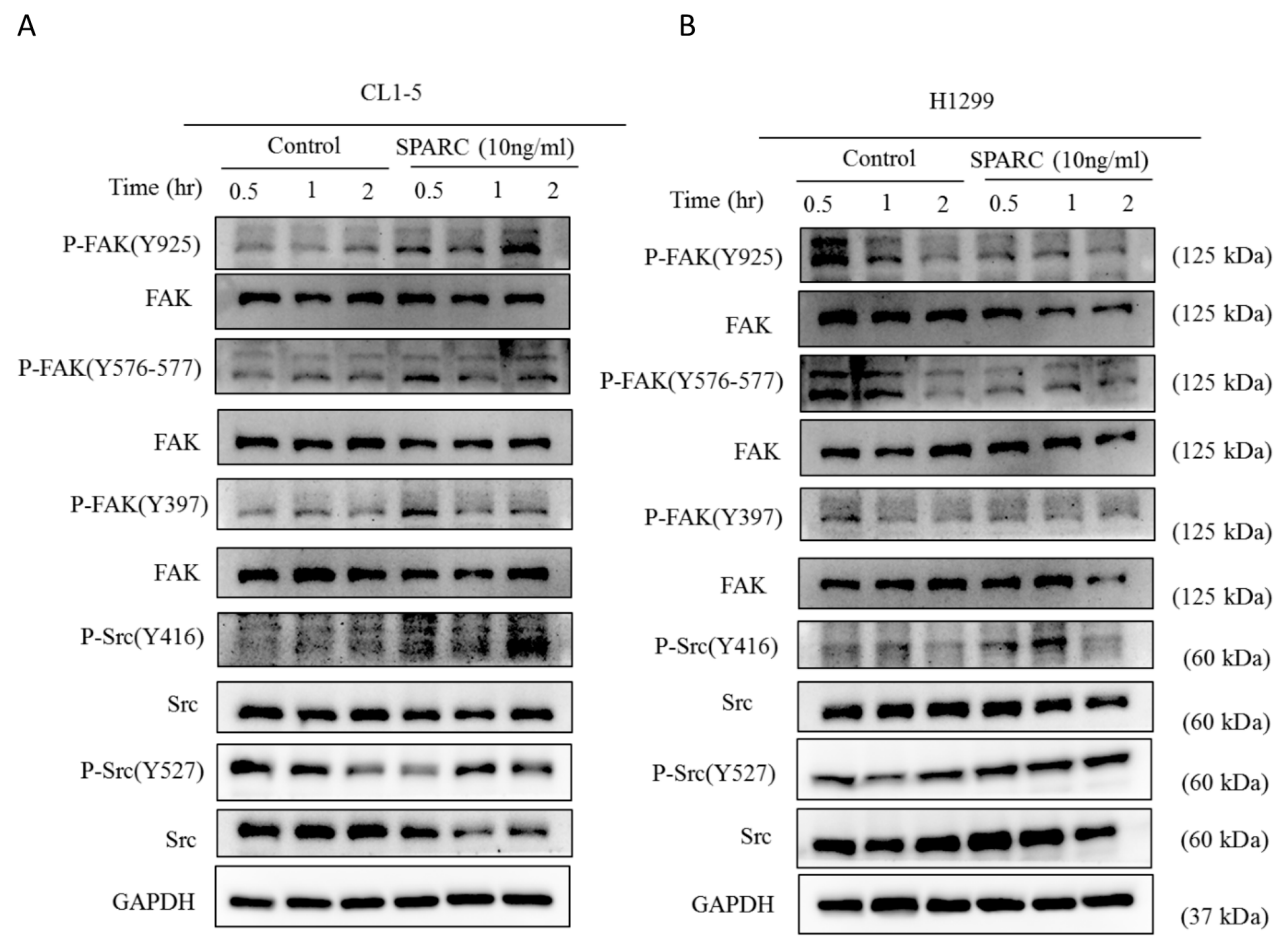
Investigation of FAK and Src signaling pathways after SPARC treatment The phosphorylation status of FAK and Src was determined in **(A)** CL1-5 and **(B)** H1299 cells after SPARC treatment.

Activated focal adhesion kinase (FAK) and Src kinase have been proven to be critical signaling pathways between the mesenchymal phenotype and extracellular components in lung cancer cells [23, 24]. In our previous study and another report, FAK is shown to be involved in regulation of mesenchymal phenotype in non-small cell lung cancer [25, 26]. In Figure 2A, SPARC treatment increased phosphorylation of Tyr925 and Tyr576-577 within FAK, and Tyr416 within Src in CL1-5. However, different phosphorylation status of FAK and Src were detected in H1299 (Figure 2B). The results suggest that FAK/Src signaling pathways are not critical regulators in SPARC-mediated EMT program in CL1-5 and H1299 cell lines.

### WNK1 is an important kinase in SPARC-induced EMT signaling pathways

**Figure 3:**
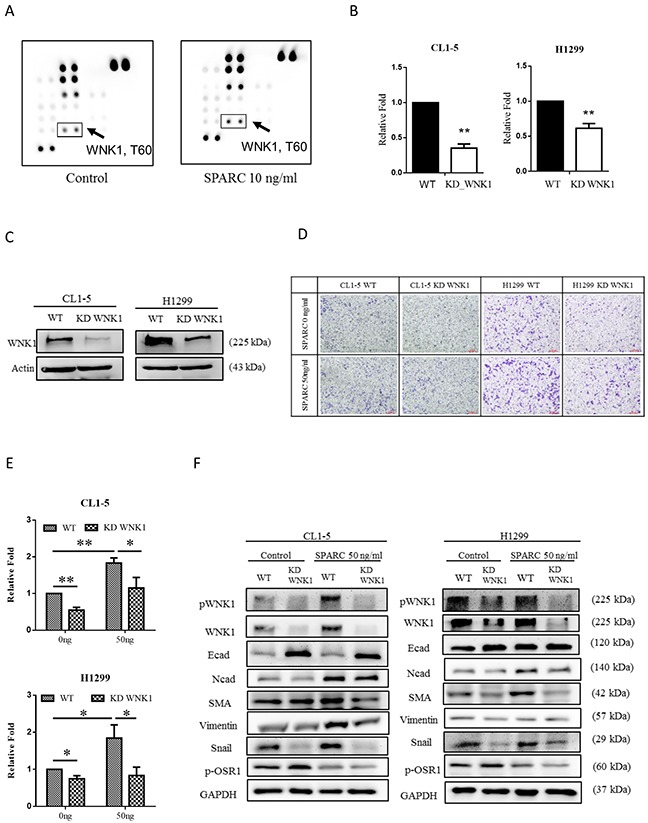
SPARC treatment induces WNK1 activation and mesenchymal phenotype **(A)** Analysis of Phospho-kinase array in control- and SPARC-treated CL1-5 cells. The arrow indicates the phospho-WNK1 on each membrane. **(B)** The mRNA expression of WNK1 and **(C)** protein expression of WNK1 after transfection of WNK1 siRNA. **(D)** Cell migration was determined by transwell migration assay. **(E)** The number of migrated CL1-5 and H1299 cells. **(F)** The effect of WNK1 knockdown in EMT signaling pathway. The error bars represent SD (t-test; * p < 0.05, ** p < 0.01, *** p < 0.001).

To further investigate if other candidate molecules are involved, the phospho-kinase array assay was performed. The phosphorylation of Thr60 within WNK1 in CL1-5 was induced after SPARC treatment (Figure 3A). Then the WNK1-knockdown of CL1-5 and H1299 were established (Figure 3B and 3C). Migration ability of CL1-5 and H1299 cells was suppressed after knockdown of WNK1 expression (Figure 3D and 3E). Phosphorylation levels of OSR1, a downstream molecule of WNK1, were suppressed in WNK1-knockdown cells (Figure 3F) [27]. In addition, knockdown of WNK1 reversed the SPARC-mediated induction of N-cadherin, smooth muscle actin and Snail, and reduction of E-cadherin (Figure 3F). These findings suggest that WNK1 signaling pathway promotes the mesenchymal phenotype in lung cancer cells.

### Snail involved in regulation of SPARC-induced EMT pathways

**Figure 4:**
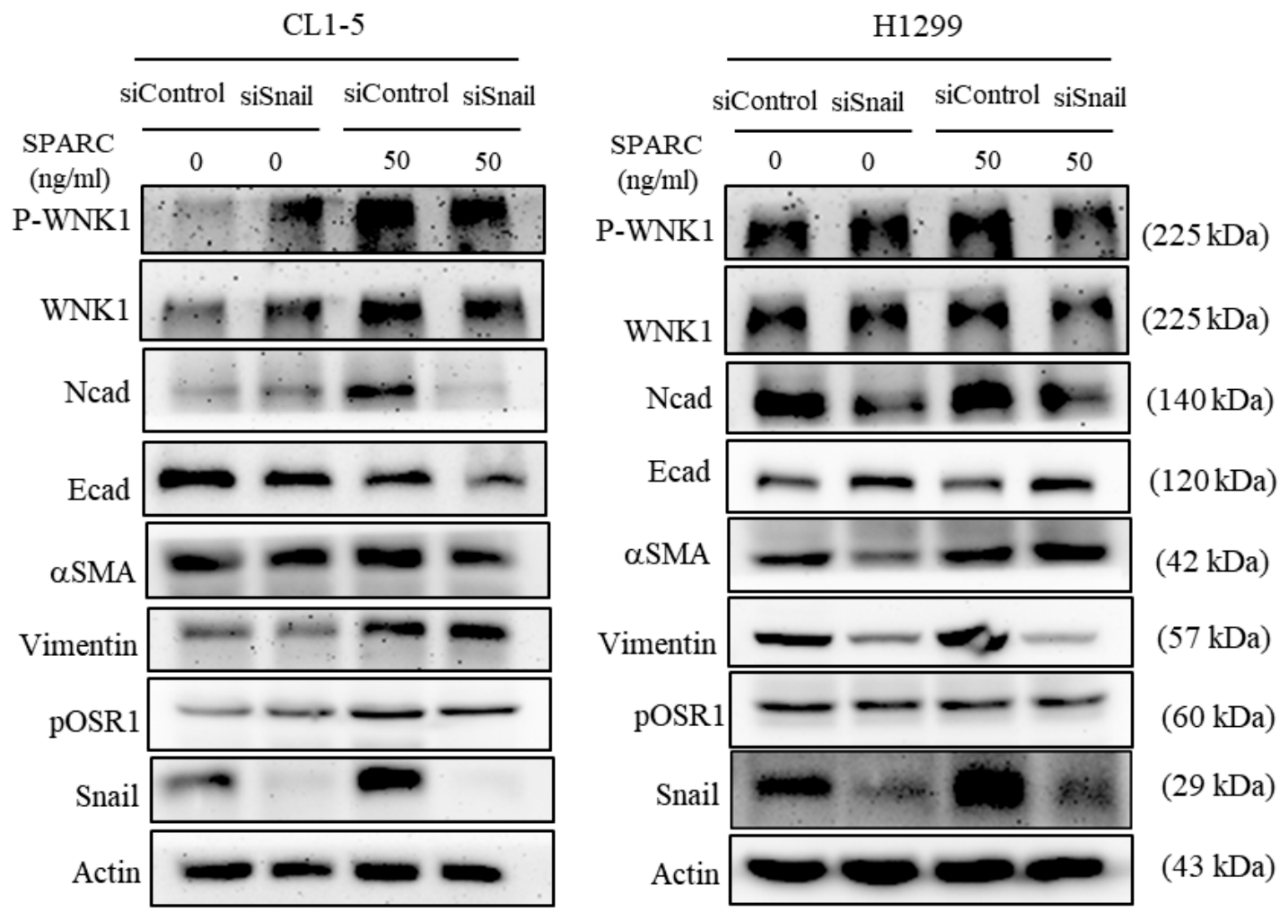
Knockdown of Snail alters SPARC-WNK1 pathway The effect of Snail knockdown insignaling pathways of EMT in CL1-5 and H1299 cells.

Because Snail overexpression induces SPARC expression in non-small cell lung cancer [19], the role of Snail in SPARC-WNK1 signaling pathway was consequently evaluated. In Figure 4, the phosphorylation of WNK1 was induced after transfection of Snail siRNA in CL1-5 cells but not in H1299 cells. Since Snail is one of the critical EMT regulators, suppression of Snail expression increased E-cadherin expression and decreased N-cadherin expression. Furthermore, Snail knockdown did not affect the SPARC treatment-enhanced WNK1 phosphorylation and the phosphorylation status of OSR1. In contrast, the SPARC-mediated effect on N-cadherin and E-cadherin was abolished in Snail-knockdown cells. The expression of smooth muscle actin and Vimentin was not altered by Snail, which suggests that N-cadherin and E-cadherin are regulated by Snail in SPARC/WNK1 signaling pathway.

### SPARC treatment induces phosphorylation of akt and WNK in a murine tumor model

**Figure 5:**
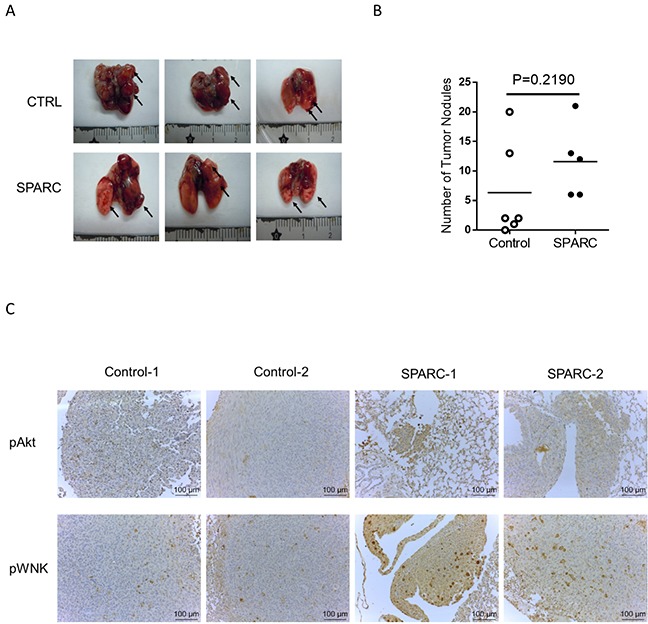
SPARC treatment induces Akt and WNK phosphorylation in a murine lung cancer model **(A)** The tumor nodules. Black arrows indicate tumor nodules. **(B)** A scatter plotter shows the number of tumor nodules. Horizontal bars indicate the mean of both groups (control mice, n=6; SPARC treated mice, n=5). **(C)** Immunohistochemistry staining of phosphor-Akt and WNK in lung tumor nodules. Antibody staining is brown, and nuclei are stained with hematoxylin. Two images of each group are shown.

We further investigated whether SPARC/WNK1 pathways *in vivo*. The effect of SPARC was examined in tumor-bearing C57BL/6 mice which were intravenously injected with the murine lung cancer cells LLC1. The images of tumor nodules and the number of tumor nodules are shown in Figure 5A and 5B. Although the number of tumor nodules in SPARC-treated mice was higher than in control mice, there was no statistically significant difference between the two groups. Because SPARC activates integrin-linked kinase/Akt pathway [28], the phosphorylation of Akt and WNK1 was determined. When compared to control mice, higher phosphorylation levels of Akt and WNK were observed in SPARC-treated mice. In Figure 5C, the image of control-1, control-2, SPARC-1 and SPARC-2 were respectively taken from independent mice. This result indicates that SPARC stimulation activates WNK and Akt pathways in lung cancer cells *in vivo*.

## DISCUSSION

SPARC is a non-collagenous ECM protein and a regulator of cell behavior [12]. In primary fibroblasts, isolated from pulmonary tissue, SPARC is essential for fibronectin-induced integrin-linked kinase (ILK) activation and downstream signaling pathways [29]. SPARC inhibits adipogenesis through activation of β-catenin pathway in adipocytes [30]. In addition, suppression of SPARC expression decreases Akt, ILK and FAK activation, and SHC/RAF/ERK signaling is also involved in SPARC-mediated invasiveness in glioma [31, 32]. In contrast, reducing stromal cell-secreted SPARC increased Akt phosphorylation in prostate cancer [33]. This evidence shows SPARC-induced diverse signaling pathways in different types of tissue. In patients with non-small cell lung cancer, higher SPARC expression and lower Kruppel-like factor 4 (KLF4) are detected in tumor tissue compared to non-tumor tissue [34]. Overexpression of KLF4 inhibits invasiveness of lung cancer cells (A549 and H322) by suppressing SPARC expression [35]. Our results reveal that knockdown of WNK1 decreases SPARC treatment-induced migration and reverses the transition of the mesenchymal phenotype in non-small cell lung cancer cell lines H1299 and CL1-5 (Figure 3D-3F). To the best of our knowledge, this is the first study describing SPARC/WNK1/Snail signaling pathway in non-small cell lung cancer.

In highly metastatic cancer, SPARC promotes migration and EMT [21, 22]. Our results also show that SPARC treatment induces migration and EMT in highly metastatic non-small cell lung cancer cell lines CL1-5 and H1299. Tumor suppressor protein p53 has been proven to regulate cancer cell invasion via Snail family transcriptional repressor 2 (SNAI2, also known as Slug) which suppresses E-cadherin expression and triggers EMT in non-small cell lung cancer [36]. Wild type p53 downregulates Slug, whereas mutant p53 results in accumulation of Slug [36]. Different p53 status have been observed in CL1-5 and H1299, in that H1299 does not express p53 protein, and CL1-5 expresses mutant p53 (R248W) [37]. It is interesting to note that E-cadherin levels are not altered by SPARC treatment in H1299 cells (Figure 1F). In Figures 3F and 4, the expression pattern of E-cadherin, Vimentin, and smooth muscle actin is not identical between CL1-5 and H1299 after manipulation of WNK1 and Snail expression. Therefore, we hypothesize that p53 might be a regulating facror of SPARC-induced EMT signaling pathways.

Immune cells, such as tumor-associated macrophages, also produce SPARC and enhance migration in murine animal models for breast cancer [38]. Sangaletti S et al. reported that in breast cancer, macrophage-produced SPARC enhances cell migration in a spontaneous tumor model but not in an experimental intravenous metastasis model [38]. The author proposed that the cancer cells might require host SPARC to leave the primary tumor but not to seed to distant organs. However, in ovarian cancer cell, knockdown SPARC expression with shRNA inhibits lung metastasis via tail vein injection of cancer cells [39]. Because the role of SPARC in the intravenous metastasis model remains unclear, the role of exogenous SPARC was evaluated *in vivo* for the present study. Intraperitoneal injection of SPARC did not significantly increase lung tumor nodules in an intravenous metastasis model, just as was found in breast cancer cells (Figure 5A and 5B)[38]. However, we have illustrated in the present study that intraperitoneal injected SPARC induces activation of Akt and WNK signaling pathways in tumor nodules. The interaction of SPARC between different cancers and their microenvironments might be an interesting subject for further investigation.

**Figure 6:**
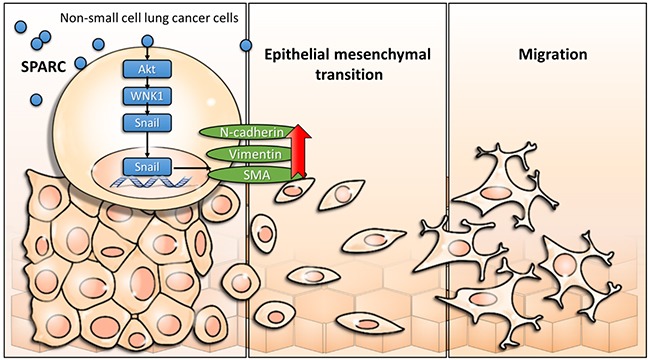
Scheme of proposed exogenous SPARC mediated signaling pathways in non-small cell lung cancer

The with no lysine (K) family are serine/threonine kinases. In humans, there are four WNK genes (WNK1, WNK2, WNK3, and WNK4) [40, 41]. WNK kinases are involved in the enhancement of cell proliferation, migration, invasion, and autophagy [40–42]. Therefore, WNK1 is a potential regulator of SPARC-mediated cell migration. In Figure 3A, SPARC induces phosphorylation of threonine residue 60 of WNK1. Akt has also been demonstrated to phosphorylate WNK1 at threonine residue 60 [43, 44]. Our previous study found that activation of Akt triggers WNK1-mediated lung cancer progression [45]. Increasing phosphorylation of Akt was observed in SPARC-treated lung tumor nodules (Figure 5C). We therefore hypothesized that SPARC-WNK1 signaling pathway is dependent on Akt in non-small cell lung cancer. Snail is also involved in SPARC treatment-induced signaling pathways. In Figure 4, silencing Snail expression reverses SPARC-induced EMT signaling pathways, but affects the phosphorylation status of WNK1. In contrast, Snail expression is suppressed after knockdown of WNK1 (Figure 3). Taken together, our results revealed a SPARC/WNK1/Snail signaling pathways, and these proposed pathways are shown in Figure 6.

In summary, this study suggests that direct SPARC treatment triggers WNK1 and induces mesenchymal phenotype. Further evidence indicates that WNK1 and Snail, but not FAK and Src, play important roles in the SPARC-mediated effect. In a murine intravenous lung metastatic model, SPARC treatment increases phosphorylation of Akt and WNK. Our study suggests that blockage of SPARC/WNK1/Snail signaling pathway could be a strategy to suppress migration of non-small cell lung cancer.

## MATERIALS AND METHODS

### Chemicals and antibodies

All chemicals were purchased from Sigma-Aldrich. Antibodies of antibodies of N-cadherin (1:1000, #610921) and E-cadherin (1:1000, #610182) were obtained from BD Transduction Laboratories; antibodies of vimentin (1:1000, #MAB3400), p-OSR1(1:1000, #07-2273), actin (1:5000, #MAB1501) and GAPDH (1:5000, #MAB374) were obtained from Millipore; antibodies of snail (1:1000, #3879S), p-FAK (Y925) (1:1000, #3284S), p-FAK (Tyr566,567) (1:1000, #3281S), p-FAK (Y397) (1:1000, #8556S), FAK (1:1000, #3285S), p-Src (Tyr416) (1:1000, #6943), p-Src (Y527) (1:1000, #2105). Src (1:1000, #2109), p-WNK1(T60) (1:1000, #4946), and WNK1 (1:1000, #4979) were obtained from Cell Signaling Technology; antibody of α-smooth muscle actin (1:1000, #A5228) was obtained from Sigma-Aldrich. The dilution fold of each antibody in western blot assay, and the catalog number of each antibody are shown in parentheses.

### Cell culture

The highly invasive human lung adenocarcinoma cell line, CL1-5, was kindly provided by Dr. Pan-Chyr Yang (Department of Internal Medicine, National Taiwan University Hospital, Taipei, Taiwan) [38], and H1299 derived from metastatic lymph node tumors was purchased from American Type Culture Collection (ATCC). CL1-5 and H1299 were maintained in RPMI 1640 media which was supplemented with 10% fetal bovine serum (FBS) and 1% penicillin-streptomycin (Life Technologies, Grand Island, NY, USA) and incubator 37°C, 5% CO_2_.

### Proliferation assay

For cell proliferation measurement, the WST-1 (4-[3-(4-iodophenyl)-2-(4-nitrophenyl)-2H-5-tetrazolio]-1,3-benzene disulfonate) method (Clontech) was used to measure cell proliferation. Briefly, 5 × 10^3^ CL1-5 or H1299 were seeded in 96-well plates overnight. Before each experiment, the medium was replaced by RPMI1640 with 0.5% FBS for 6 hours. The proliferation rate was determined at a wavelength of 450 nm on a microplate spectrophotometer (Power Wave X340, BioTek, USA).

### Transwell migration assay

Cell migration assay was performed in QCM™ 24-well Cell Migration Assay and Invasion System uncoated 8  μm pore size polycarbonate membranes (Millipore), according to the manufacturer's instructions. Briefly, 3 × 10^5^ CL1-5 or H1299 cells were seeded into 24 well insert in 300 μl of serum free medium, while 500 μl medium with 10% FBS was in the lower chamber. After 24 hours, the bottom surface of the membrane was fixed in 4% formaldehyde solution, followed by 1% crystal violet staining. Cells on the upper surface were removed by a cotton swab after membrane washing. The bottom of the membrane was then visualized using the Olympus inverted microscopes at 100X magnification. Four random fields of view were counted and the relative fold of migration in each group was compared to the 0 ng/ml SPARC treated group.

### Western blot

Cells were lysed in radioimmunoprecipitation lysis buffer (RIPA) (Millipore) with protease inhibitor cocktail (Millipore), and the total cell lysate was collected after centrifugation at 4°C, 12000 x g for 15 minutes. Quantification of protein concentration was made by BCA protein assay kit. Equal amounts of protein was loaded into each lane of 10% SDS-PAGE and transferred to polyvinylidene difluoride membranes (Millipore). Membranes were blocked with 5% skim milk in Tris-buffered saline with Tween-20 (TBST) buffer, and sequentially incubated with primary antibodies overnight at 4°C, and secondary antibodies for 1 hour at room temperature. The results were analyzed using an Alpha Innotech FluorChem FC2 imaging system (ProteinSimple; Bio-Techne, Minneapolis, MN)

### Quantitative polymerase chain reaction (QPCR)

Total RNA was extracted from cells using TRIzol reagent (Invitrogen; Thermo Fisher Scientific, Inc.) according to the manufacturer's protocol. PrimeScript RT reagent kit (Clontech Laboratories, Inc., Mountainview, CA, USA) was used for reverse transcription of complementary DNA (cDNA) from mRNA, also according to the manufacturer's protocol. PCR was performed using the following primers: WNK1 forward, 5′-CTTTATCACCGGCCCTACTG-3′ and reverse, 5′- TAGCCATCTCAAGCATGCAC-3′; GAPDH forward, 5′-GAGTCAACGGATTTGGTCGT-3′ and reverse, 5′-TTGATTTTGGAGGGATCTCG-3′. PCR was performed on a StepOne Plus Real-Time PCT System (Applied Biosystems; Thermo Fisher Scientific, Inc.) using the Fast SYBR Green Master Mix (Applied Biosystems; Thermo Fisher Scientific, Inc.). Relative mRNA expression levels were normalized to the expression level of GAPDH using the 2^−ΔΔCt^ method.

### Phospho-kinase array

Six hundred μg protein lysate of CL1-5 cells, which were respectively treated with or without SPARC (10 ng/mL) for forty-five minutes or were collected. Protein samples were incubated with Human Phospho-Kinase Array Kit (Proteome Profiler Array, ARY003B, R&D Systems) in accordance with the manufacturer's instruction. The images were adapted on an imaging capture system (Alpha Innovation).

### Knockdown of WNK1 and snail

For knockdown of WNK1 expression, WNK1 shRNA (targeting sequence: 5′-CCGCGATCTTAA ATGTGACAA-3′) was obtained from RNAi, miRNA, miRNA sponges, and CRISPR/Genomic Research Center, Academia Sinica (Taipei, Taiwan). CL1-5 and H1299 cells were transfected with 1μg of WNK1 shRNA via lipofectamine 2000 (Invitrogen), in accordance with the manufacturer's instructions. WNK1-knockdown stable clones were selected and maintained in medium containing 2 μg/ml puromycin. ON-TARGETplus siRNA, containing four siRNAs targeting Snail (catalog no. L-010847–01) and control siRNA (catalog no. D-001810-10-05) were purchased from Dharmacon. CL1-5 and H1299 cells were transfected with siRNA (final concentration was 10 nM) by DharmaFECT transfection reagent 1 (Dharmacon), according to the manufacturer's instruction. Knockdown efficiency of Snail siRNA was evaluated by QPCR and western blot assay 48 hours post transfection.

### Animal tumor model

Use of all animals in this study was approved by the Animal Care and Use Committee at Kaohsiung Medical University. Six-week-old male C57BL/6 mice (BALB/cByJNarl) were purchased from the National Laboratory Animal Center (Taiwan) and maintained in pathogen-free conditions in the Laboratory Animal Center of Kaohsiung Medical University Hospital. Mice were intravenously injected with 5 × 10^5^ cells LLC1 cells in 200 microliter. One day after tumor cells injection, mice were treated with mouse SPARC (25 microgram / 100 microliter PBS / mouse, obtained from R&D Systems, Minneapolis, MN) or PBS (control mice) via intraperitoneal injection twice a week. Mice were sacrificed and the tumor nodules counted at 24 days after tumor injection. Tumor samples were paraffin-embedded and then sliced into 5 μm thickness. The phosphorylation status of Akt and WNK was stained (1:100 dilution).

### Statistical analysis

All bar graphs and statistics were performed by GraphPad Prism 7 (GraphPad Software, San Diego, CA). Student's t-test was used for analysis of difference between the two groups. P-value < 0.05 was considered to indicate a statistically significant difference.
